# Local Anesthesia for Port Catheter Placement in Oncology Patients: An Alternative to Landmark Technique Using Ultrasound-Guided Superficial Cervical Plexus Block—A Prospective Randomized Study

**DOI:** 10.1155/2019/2585748

**Published:** 2019-07-31

**Authors:** Hakan Akelma, Fikret Salık, Mustafa Bıçak, Meral Erdal Erbatur

**Affiliations:** Health Sciences University, Gazi Yaşargil Training and Research Hospital, Anesthesiology and Reanimation Clinic, Diyarbakir, Turkey

## Abstract

**Background/Objectives:**

Most patients that require port operation have experienced severe pain due to multiple surgeries in the past. Therefore, these patients have fear of pain before the procedure. This study aims to compare superficial cervical plexus block (SCPB) with local infiltration anesthesia in terms of comfort.

**Methods:**

100 cancer-diagnosed patients were divided into two groups. The first group, the landmark technique with local infiltration anesthesia, was used for intravenous entry (*Group LM, n = 50*). The second group, USG, was used for venous entry with SCPB as anesthesia* (Group US, n = 50)*. The type of local anesthesia, port placement technique, duration of the procedure, number of procedures, complications, visual analog scale (VAS) score, and surgeon's satisfaction with the procedure were recorded.

**Results:**

It was established that an average of 1.7 and 1.1 attempts was conducted in Groups LM and US, respectively (*P* = 0.010). VAS scores were found to be 4.04 in Group LM and 2.62 in GroupUS (*P* = 0.001). Surgeon satisfaction was 1.96 in GroupLM and 2.38 in GroupUS (*P* = 0.014). The mean duration of the procedure was 22.10 minutes in GroupLM and 43.50 minutes in GroupUS (*P* = 0.001). Complication rates were observed in 1 patient in GroupLM and 9 patient in GroupUS (*P* = 0.040).

**Conclusions:**

In the patient group with a high level of pain and anxiety port catheter placement using USG and SCPB, supported by routine sedation, provides better comfort for both patient and surgeon.

## 1. Introduction

Infusion therapy through central venous subcutaneous port catheters (CVSPC) is a more favorable alternative than the peripherally placed central catheter or tunnel catheters to peripheral vessels [1]. These catheters that provide safe and easy vascular access in repetitive drug applications and are fully subcutaneous have become a standard practice to provide long-term venous access to chemotherapy, antibiotherapy, or parenteral nutrition in recent years. Other advantages are that they have a low risk of infection, they do not have any parts disturbing the patient on the surface, and they do not prevent physical activity of the patient [2]. When subcutaneous venous ports are placed, the antecubital veins, axillary vein, internal jugular vein (IJV), subclavian vein (SV), or femoral vein may be preferred [3] as a place of interference for venous cannulation. While performing this procedure, local anesthesia is commonly used for venipuncture. In recent years, superficial cervical plexus block (SCPB) has been used for IJV or SV applications. Despite the widespread use of local infiltration anesthesia, SCPB is now frequently used while opening and tunneling for the port. During CVSPC placement and its use, major or minor complications, such as hemothorax, pneumothorax, arrhythmia, malposition, thromboembolism, arteriovenous fistula, nerve injuries, infection, or catheter ejection, may develop [4].

To our knowledge, studies comparing the use of local infiltration anesthesia for port catheter placement in the jugular vein and SVs and SCPB with ultrasonography (USG) have not been conducted yet. This study aims to compare and discuss the vein entry after SCPB using the USG-guided CVSPC procedure with the landmark technique using local infiltration anesthesia in terms of the number of trials, success rate, surgical comfort, pain scores, malpositions, and complications.

## 2. Materials and Methods

### 2.1. Patient Selection

**Figure 1:**
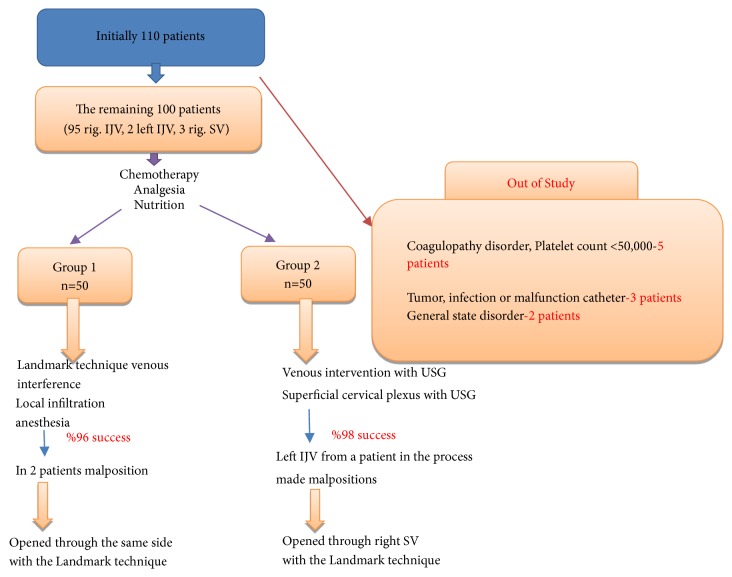
Patient selection, grouping, anesthesia method, and results.* IJV*, internal jugular vein;* SV*, subclavian vein;* USG*, ultrasonography.

This study was approved by local ethics committees where appropriate and performed in accordance with the Helsinki Declaration. This prospective randomized observational study was designed with regard to CVSPC placement in 100 cancer-diagnosed patients to receive chemotherapy, on approval of the ethics committee of our hospital, dated 12-28-2018, and numbered 190. The nurse and doctor in the oncology and palliative care clinic informed the patients who would undergo port placement and their relatives regarding the procedure and their written and verbal approvals were obtained. Patients were randomly divided as two groups with 50 patients in each group. Cancer patients were randomly sent to the physician's room by two experienced (two assistant professors) physicians who would perform the procedure by the clinical nurse. Independent two physicians applied the procedure to each group and recorded the results. Both physicians independently applied their own method and recorded the results. All the oncological and palliative care patients with no general condition disorder and those who needed chemotherapy and permanent port catheter for analgesia and nutritional purposes were included in the study (Figure 1).

### 2.2. Patient Exclusion Criteria

Patients with coagulopathy, thrombocytopenia (below 50,000), infection at the site of intervention, tumor, and general condition disorder were not included in the study. In total, 10 out of 110 applicants were excluded from the study (Figure 1).

### 2.3. Anesthesia Preparation and Surgical Approach

The patients were divided into two groups according to the types of venous access and local anesthesia, which are routinely applied as port placement methods at our hospital. In the first group, local anesthesia was applied to the venous access site, supraclavicular landmark entry point, port pocket area, and catheter subcutaneous passage line. The port was inserted after the central venous puncture using the landmark technique (*Group LM, n = 50*). In the second group, first, the port was inserted using USG after the application of SCPB and then central venous puncture using USG was conducted (*Group US, n = 50*; Figure 1).

All procedures were performed with local anesthesia or SCPB under intravenous sedoanalgesia in the operating room. For sedation, 0.15–0.20 mg/kg midazolam (Dormicum, Roche, Germany) and 0.5–1 *μ*g/kg fentanyl citrate (Fentanyl Citrate 50 *μ*g/mL 10 mL ampoule, Abbott Laboratories, IL, USA) were administered intravenously. Electrocardiography, peripheral oxygen saturation (SpO_2_), and noninvasive blood pressure monitoring were performed. Oxygen at a flow rate of 2 L/min was administered using nasal cannula. During the intervention, the patients with pain, in both groups, were administered 0.5–1 *μ*g/kg fentanyl citrate (Fentanyl Citrate 50 *μ*g/mL 10 mL ampoule, Abbott Laboratories, IL, USA) for additional local anesthetic infiltration and sedoanalgesia. Initially, right IJV was preferred for the procedure in all patients. The left IJV was considered if the right IJV was obstructed or an obstacle was to be introduced. Right SV was used in the case of bilateral disability, and contralateral IJV was preferred in patients with a history of mastectomy.

### 2.4. Venous Access and Local Anesthesia Using Landmark Technique

**Figure 2:**
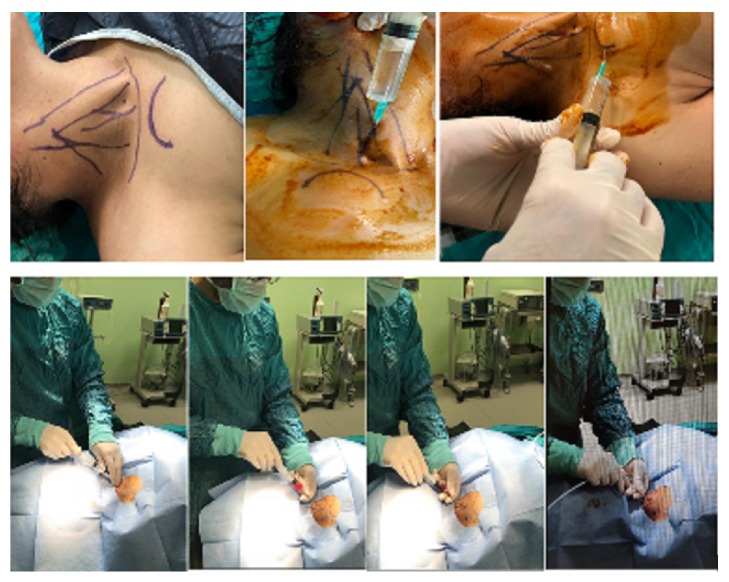
Three-point local anesthesia technique and venous intervention through landmark technique.

In this technique, once the area to be treated was covered in a sterilized manner, 10-mL 1% lidocaine (Jetoka Simplex, Adeka, Istanbul, Turkey) was administered to the pocket area where the port was placed, and 10-mL 2% prilocaine (Citanest 2% vial, AstraZeneca, Cambridge, UK) was used for the venous intervention site and tunnel; therefore, a total of 20-mL local infiltration anesthesia was applied to three regions. Accordingly, the first choice for port catheter placement is the right IJV, which is mostly preferred for central venous catheterization through the supraclavicular route [5]. The anatomical points for venous catheterization were the apex of the triangle formed by the clavicular and sternal legs of the sternocleidomastoid (SCM) muscle [6]. After the patient was placed in the supine position, the head of the bed was slightly inverted to ensure distraction of the veins and prevent air embolism. The needle was then entered into the skin at an angle of 30–40 degrees. It was directed toward the nipple on the same side. The needle was passed through the skin by 2–3 cm and the needle guide was placed after the aspiration of venous blood into the syringe, and the procedure was continued according to the Seldinger technique [7] (Figure 2).

### 2.5. USG-Guided Venous Entry and Superficial Cervical Plexus Block Technique

**Figure 3:**
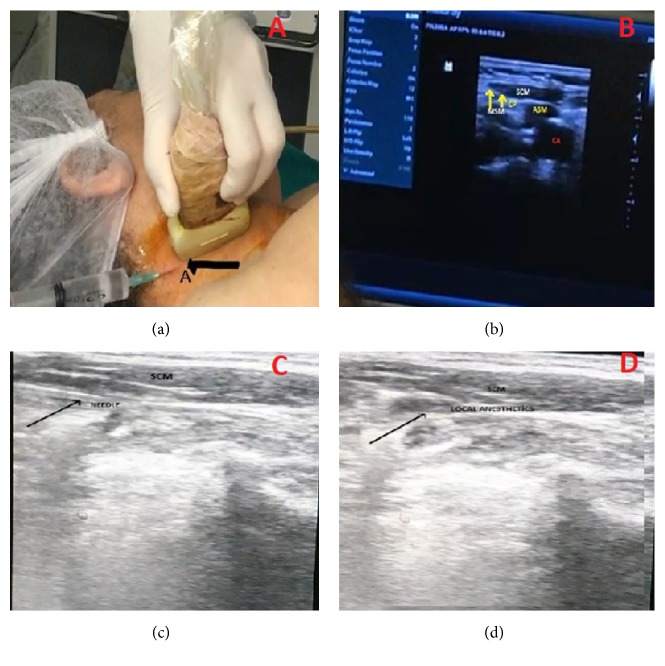
(a) The entry point through SCM midline lateral for superficial cervical plexus block (b).* SCM*, sternocleidomastoid muscle,* CA*, carotid artery,* ASM*, anterior scalene muscle, and* MSM*, middle scalene muscle. (c) Needle and SCM (d) local anesthetic distribution and SCM.

**Figure 4:**
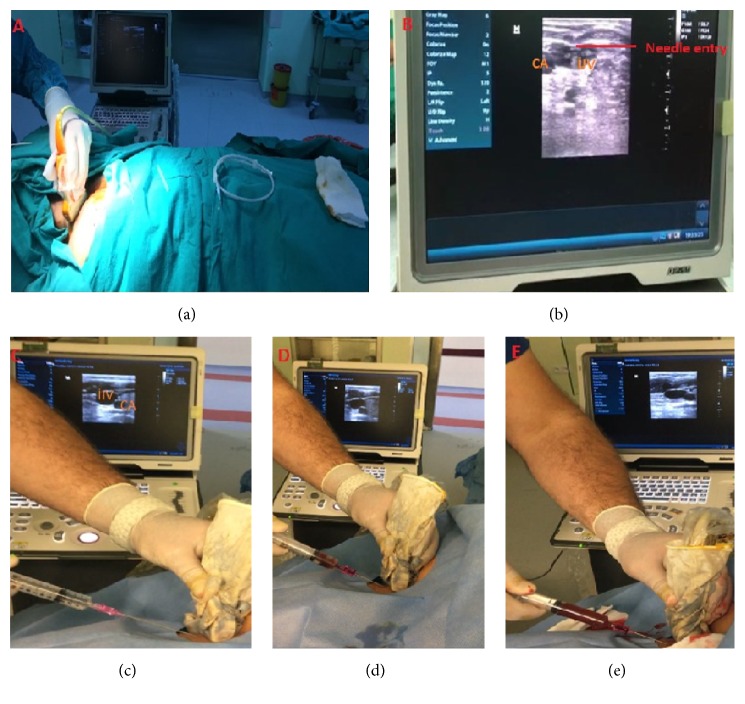
Venous intervention through USG method. Jugular vein is out of plane view. b, c, d, and e represent a needle that appears as a hyperechoic point on the ultrasound screen within the lumen of the jugular vein and entry appears in the blood of the injector.* IJV*, internal jugular vein;* CA*, carotid artery.

To determine the vein diameter and patency of the patient, a neck vein examination was performed using USG before the procedure. After the process, the area was disinfected and covered with a sterile drape. Asepsis was induced by wrapping a sterile gel or probe with a sterile surgical glove. SCPB was introduced using 10 mL of 2% prilocaine (Citanest 2% vial, AstraZeneca, Cambridge, UK) applied to the lower part of the SCM fascia after the image was obtained from the midline of the SCM muscle with the help of USG (Mindray DP-50 Digital Portable US Machine, Shenzhen, China) (Figure 3). Then, with the help of USG-guided catheterization, the transducer (5–10 MHz) was placed on the lateral side of the probe and the ultrasound probe was applied at an angle of 90 degrees to the long axis of the targeted vessel. The vein was centered in a slight motion at the center of the screen, and the needle was carefully advanced under the ultrasound guidance until the anterior wall was pierced and venous blood was aspirated into the syringe. After documenting the aspiration of venous blood, the procedure was continued according to the Seldinger technique. The presence of the guide wire in the vein along the long axis was confirmed using USG (Figure 4).

The vein catheterization point and 0.5-cm lateral and medial parts were dilated for the port placement procedure after venous entry using the respective methods.

**Figure 5:**
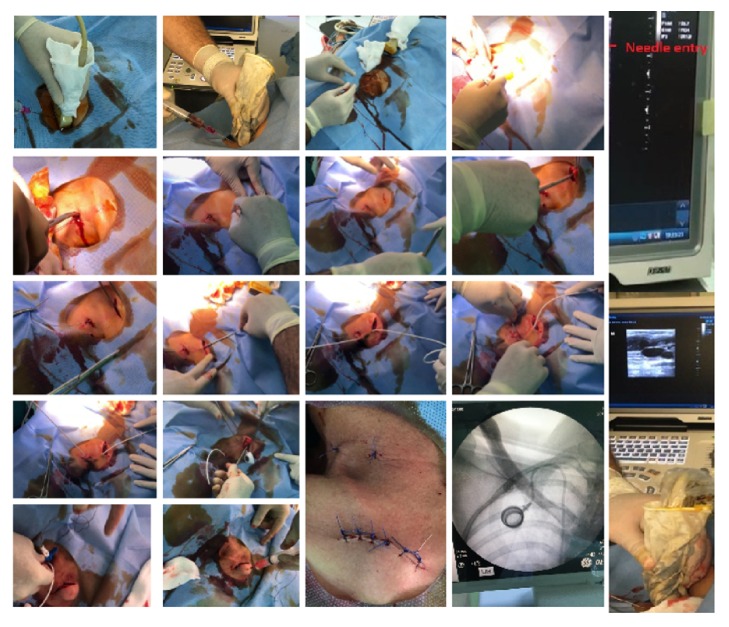
Port opening from binary tunnel using bridging method.

Further, for the port pocket, 2–3 cm distal to the clavicle midline was used in both patient groups. Tunneling was performed using bridges through the two dilated areas (Figure 5). The port pocket was then closed with sutures after checking whether the port works. All patients were evaluated by C-arm scopy and posteroanterior chest X-ray. The site of the procedure was then dressed, and the patient was moved out of the operating room.

### 2.6. Postoperative Procedure

All patients were followed up for at least 30 minutes in the postanesthesia care unit. In the absence of complications, they were transferred to the clinics. The patients who were hospitalized for 1 day were followed up for 2 hours with regard to hematoma, swelling, and edema. After the procedure, port care was demonstrated to the patients and their relatives by the responsible nurse in the clinics. Information regarding possible issues and complications was provided to the patients. The patients, who were prescribed by the clinician, were discharged 1 week later with the recommendation of suture, infection, and hematoma control.

### 2.7. Data Collection and Evaluation

During the study, the demographic data, clinical diagnosis, type of local anesthesia, port placement technique, duration of the procedure, localization of the procedure, number of procedures, arterial puncture, hematoma, arrhythmia, shortness of breath and pneumothorax, presence of malposition, additional local anesthesia and sedation requirement, visual analog scale (VAS) score, and surgeon's satisfaction with the procedure were recorded prospectively (a range between 0 and 3 is used; a score of 3 indicates that you are very satisfied and 0 indicates that you are not at all satisfied). The malpositions were recorded by verifying the port catheter with C-arm scopy.

### 2.8. Statistical Analyses

Statistical analysis was performed using SPSS 20 (SPSS Inc., Chicago, IL, USA). In the evaluation, numerical data were expressed as mean ± standard deviation (SD), median, range (smallest to largest), and categorical data were represented in percentage (%). The central tendency was expressed as the mean (SD) if the variables were normally distributed. The tools were compared using independent or paired Student's* t*-test and chi-square test.* P* values of less than 0.05 were considered to be significant.

## 3. Results

The mean age of the patients in the study was 54.28 years in Group LM and 53.14 years in Group US. Of the 100 patients who received CVSPC, 59 (*n* = 59, 59%) were male and 41 (*n* = 41, 41%) were female. In total, 95 patients received catheters using right IJV (Table 1).

**Figure 6:**
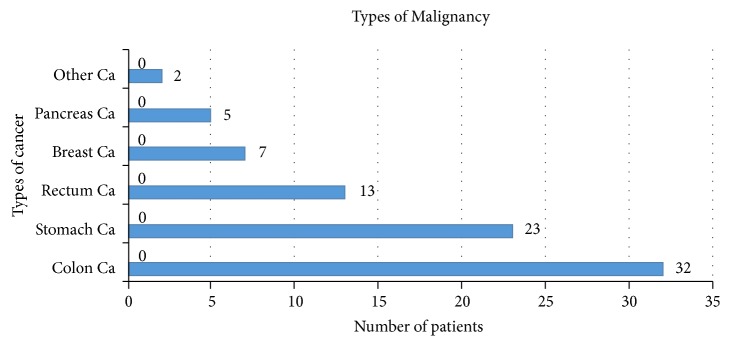
Malignancy type.

When the inserted catheters were classified in terms of malignancy types, there were 32 patients with colon cancer in the highest proportion (32%). Second, 23 patients (23%) had gastric cancer. The other types of cancer are provided in Figure 6.

In Group LM, 4 (8%) of 50 patients who underwent local anesthesia required additional local anesthetic and sedoanalgesic medication, and in Group US, 5 (10%) of 50 patients who underwent SCPB with USG required additional local anesthetic and sedoanalgesic medication. Two groups were found to be statistically similar (*P* = 0.727; Table 1).

When the groups were compared in terms of the number of trials, an average of 1.7 and 1.1 attempts was conducted in Groups 1 and 2, respectively. A statistically significant difference was found between the groups (*P* = 0.010).

When the groups were compared regarding VAS scores, average values of 4.04 and 2.62 in Groups 1 and 2, respectively, was determined. There was a statistically significant difference between the groups (*P* = 0.001).

In terms of the surgeon's satisfaction with the procedure, an average of 1.96 in Group LM and 2.38 in Group US was established. A statistically significant difference was found between the groups (*P* = 0.014).

The mean duration of the procedure was 22.10 minutes in Group LM and 43.50 minutes in Group US. There was a statistically significant difference between the groups (*P* = 0.001) (Table 1).

When the complication rates between the groups were compared, arterial puncture developed in four patients in Group LM, whereas no arterial puncture was observed in Group US (*P* = 0.041). Hematoma developed in three patients in Group LM and in one patient in Group US (*P* = 0.307). In addition, pneumothorax was detected in two patients in Group LM, but no pneumothorax was observed in any of the patients in Group US (*P* = 0.153). Complications were observed in 9 patients (18%) in Group LM and 1 patient in Group US (*P* = 0.040). There was a statistically significant difference between the groups in terms of complication rates (*P* = 0.040) (Table 2).

## 4. Discussion

Mainly, this study aimed to compare SCPB with local infiltration anesthesia using USG during CVSPC placement in terms of comfort and to determine the superiority of USG during venous access, in addition to these two methods. The study revealed that, during CVSPC placement procedure for the chemotherapy recipients, SCPB application with USG using a single injection would be superior compared with local infiltration anesthesia application from multiple points at the intervention site. Furthermore, it required less number of attempts and provided more patient comfort with an acceptable VAS score. In addition, vascular puncture using USG was observed to superior compared with the landmark technique in terms of acute complications during CVSPC placement.

Port catheters have marked advantages compared with other venous catheters because they do not restrict the patient's daily activities and cause a low rate of infection. The most common indication for CVSPC is the provision of a central venous pathway for long-term chemotherapy in oncology patients. Antecubital veins may be preferred over axillary vein, IJV, SV, or femoral vein as a site for venous entry [3, 8]. Despite the widespread use of radiological imaging techniques, IJV is recommended because of the low risk of pneumothorax, hematoma, and venous thrombosis [9–14]. Although IJV is preferred, SV is used in some cases [9, 15, 16]. The use of the femoral vein is not recommended, unless it is necessary, because of the high risk of infection and the difficulty of maintenance [13]. In this study, the right IJV was used with 95 of total patients (95%), in accordance with the literature. The SV procedure was not preferred because of the high risk of pneumothorax, unless it was required.

**Figure 7:**
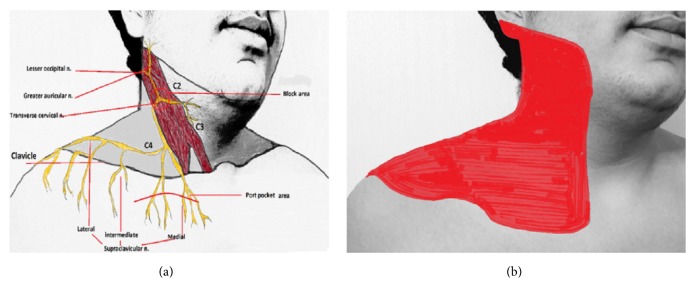
(a) Anatomy of the cervical plexus. The cervical plexus is seen emerging behind the posterior border of the sternocleidomastoid muscle. (b) Expected sensory distribution of cervical plexus blockade.

Most patients who required port operation have experienced severe pain due to the multiple surgeries that they have undergone. Therefore, their anxiety levels were very high. Some patients were anxious enough to demand general anesthesia. The most common reason for this is the fear of pain before the procedure. The aim was to administer sedoanalgesia with midazolam and fentanyl prior to the procedure to reduce the pain and related anxiety and to provide local anesthesia with minimal pain. In most of the previous studies, local infiltration anesthesia was applied to the pocket region of the anterior chest wall, which would be the site for both central venous cannulation and port placement [13, 17]. However, in recent years, SCPB has been introduced for central venous catheter procedures, neck/thyroid surgery, carotid endarterectomy, and clavicle circumference measurements. Anatomically, the cervical plexus is formed by anterior divisions of four upper cervical nerves (C1–C4). The SCM muscle forms “a roof” over the superficial cervical plexus nerves. The roots converge to form the four terminal branches (smaller occipital, great auricular circumference, transverse cervical, and supraclavicular nerves) and appear behind the posterior border of the SCM muscle. They spread under the skin through the back of the head auricula, neck, clavicle circumference, and superficial branch structures leading to the shoulder. They form the sensory fibers of these regions [18–20] (Figures 7(a)–7(b) show the domain of the plexus) (Figure 7). Çiftci et al. [19] reported that when SCPB was used prior to the insertion of a hemodialysis catheter into either IJV or SV, patients had low pain scores during the procedure and the block was successful and did not require additional sedoanalgesia in any patient. Ben Ho et al. [20] reported that they performed SCPB in the cervical fractures, paracervical muscle spasm, acromioclavicular joint injuries, and rotator cuff disorders in the emergency department and obtained significant analgesic results. Furthermore, they stated that no additional sedoanalgesia was required. In this study, four (8%) and five (10%) patients required additional local anesthetic and additional sedoanalgesia in Groups LM and US, respectively (*P* = 0.727). The statistical comparison of the groups was similar. Low VAS scores were obtained in Group US (*P* = 0.001). Similarly, the surgeon's satisfaction with the procedure was found to be higher and statistically significant in Group US (*P* = 0.014). In the results, the requirement of additional local anesthetic and sedoanalgesic medication in the CVSPC procedure was similar. However, the VAS scores, which are the main determinant of patient comfort, were significantly lower in the SCPB group with USG, which would have better results in various applications. The difference in the VAS scores may be because the pain experienced during local infiltration anesthesia is more severe than that in SCPB with USG, accompanied by a single injection.

CVSPC placement, which is based on central venous access, must be performed with USG. According to reports, USG reduces the risk of complications, such as arterial puncture from vascular injury and secondary hematoma, and most importantly, it reduces the possibility of pneumothorax/hemothorax compared with the landmark technique [3, 8, 10, 21–27]. In previous studies, it has been observed that the rate of pneumothorax during central venous intervention varies between 0.35.6% [11, 13, 28]. In the study by Bruzoni et al. [24] pneumothorax was detected in two patients in the landmark technique group; however, none of the patients in the USG group had pneumothorax, although one patient developed hemothorax. In the present study, although six (12%) patients in Group LM had complications, only one (2%) patient in Group US had complications (*P* = 0.040). This situation is statistically significant and similar to that in previous studies. Thus, it shows the superiority of USG use. Although pneumothorax developed in two (4%) patients in Group LM, it was not observed in any patients in Group US (*P* = 0.012). When one patient had severe pneumothorax, tube thoracostomy was performed. The other three patients had mild dyspnea, oxygen requirement, and chest pain because of minimal pneumothorax. Nasal oxygen was followed by regular chest radiography without the requirement for tube thoracostomy. All patients in this study were discharged with follow-up and treatment advice.

**Figure 8:**
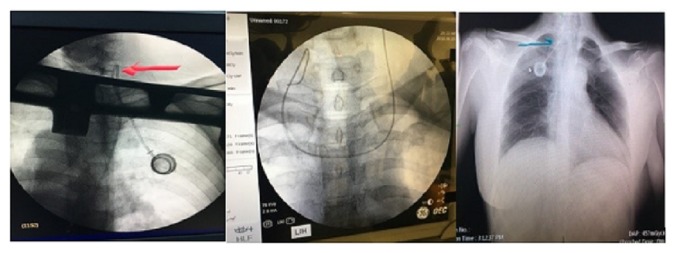
Malposition and flection development of in sample patients.

In addition to the major advantages of USG use during SVPK placement, the use of other imaging methods is important for the correct termination of the procedure. In a study reported by Schummer et al. [29] 1794 port catheters were placed using the Seldinger technique and malposition was encountered in 121 (6.7%) patients. Miccini et al. [14] reported that they encountered malposition in four (1.4%) patients. In this study, in one patient who underwent right IJV (Group LM) after guide-wire placement, the guide wire was progressing to the innominate vein on the same side; furthermore, in one patient who underwent left IJV (Group US), the guide wire progressed to the innominate vein on the opposite side. In both patients, success was achieved by changing the intervention site. In three patients, the catheter made malposition and flection, and in one patient, the end of the catheter advanced and progressed to the right ventricle. Catheter placement was corrected using the necessary intervention in these patients (Figure 8: malposition and flection patient samples).

This study had some shortcomings. First, the bed capacity was only 36 in the palliative care and medical oncology units. The number of opened ports in this study is limited because of the less number of beds available. Second, late-term complications were not evaluated. Further randomized controlled trials with larger prospective patients are required to contribute to the literature.

## 5. Conclusion

CVSPC placement causes high levels of anxiety in patients who have undergone multiple surgeries and/or interventions. Thus, some patients are apprehensive and request for general anesthesia. Therefore, sedoanalgesia support and CVSPC placement with USG and SCPB can provide better comfort for the patients and surgeon.

USG use for venous intervention during CVSPC placement can reduce the rate of complications.

USG use during venous intervention provides ease of fluoroscopy use during and after the procedure and helps in correct CVSPC placement.

## Figures and Tables

**Table 1:** Demographic and clinical data of the groups (mean ± SD).

	GROUP 1 (n = 50)	GROUP 2 (n = 50)	*P* Value
Age	54.28	53.14	*P > 0.05*
Gender (F/M)	25/25	34/16	*P > 0.05*
Place of Intervention(Jugular)	49(98%)	48(96%)	*P > 0.05*
Additional Local	4(8%)	5(10%)	*P = 0.727*
Additional Sedoanalgesia	4(8%)	5(10%)	*P = 0.727*
Number of Trials	1.7	1.1	*P = 0.010*
VAS	2.62	4.04	*P = 0.001*
Surgical Satisfaction	1.96	2.38	*P = 0.014*
Processing Time (min)	22.10	43.50	*P = 0.010*

*VAS*, visual analog scale.

**Table 2:** Number of complications between groups (mean ± SD).

	GROUP 1 (n = 50)	GROUP 2 (n = 50)	*P* VALUE
Artery Puncture	4(8%)	0(0%)	*P = 0.041*
Hematoma	3(6%)	1(2%)	*P = 0.307*
Pneumothorax	2(4%)	0(0%)	*P = 0.153*
Complication	9(18%)	1(2%)	*P = 0.040*

## Data Availability

The data used to support the findings of this study are available from the corresponding author upon request.
